# Effect of Excitation Wavelength in Single‐Molecule Photochemistry of Terrylene

**DOI:** 10.1002/cphc.202400996

**Published:** 2025-01-26

**Authors:** Rana Mhanna, Julia Berger, Matthias Jourdain, Stephan Muth, Roger Jan Kutta, Gregor Jung

**Affiliations:** ^1^ Department of Biophysical Chemistry Saarland University 66123 Saarbrücken Germany; ^2^ Institute of Physical and Theoretical Chemistry University of Regensburg 93053 Regensburg Germany

**Keywords:** fluorescence spectroscopy, single-molecule studies, singlet oxygen, cycloaddition

## Abstract

The reaction of terrylene in *p*‐terphenyl with molecular oxygen is reinvestigated by TIRF‐microscopy with λ_exc_=488 nm or λ_exc_=561 nm and 488 nm. A similar range of fluorescent products is obtained under both experimental conditions with a reaction quantum yield Φ_r_>10^−7^ for those molecules which undergo the photoreaction. The majority of these oxygen‐susceptible molecules reacts via an electronically relaxed, dark intermediate, presumably an endoperoxide, with a lifetime of <t_off_>~20 s. From this time constant, an activation energy *E*_A_<0.8 eV is estimated for the transition from the intermediate to the final product, the diepoxide, which nicely agrees with values calculated for the terrylene‐derivative TDI. However, ~20 % of all reacting molecules at λ_exc_=561 nm and even ~40 % at λ_exc_=488 nm show an immediate change of the fluorescence colour within the time resolution of the experiment, bypassing any dark intermediate. Based on this experimentally observed impact of the excitation energy and the lack of relevant excited‐state absorption, we hypothesize that oxygen forms a complex with ground‐state terrylene which then undergoes a quasi‐unimolecular reaction in the excited‐state before vibrational relaxation takes place.

## Graphical abstract

Single‐molecule fluorescence microscopy of terrylene shows changes of the fluorescence color from yellow to green along with its photooxidation. The reaction path circumventing the endoperoxide as intermediate among the bifurcated reaction mechanism is boosted with short wavelength excitation and hints to a preformed complex of the aromatic compound with oxygen.

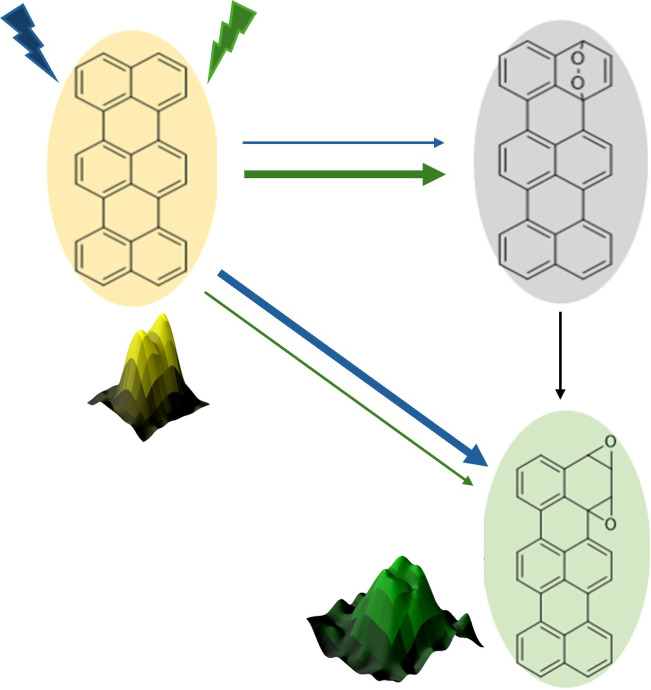

## Introduction

Single‐molecule chemistry (SMC) allows for the study of a reactive system at the molecular scale through real‐time observation and precise measurement of observables, which cannot be revealed in bulk.^[1]^ One effective approach involves tracking the temporal evolution of fluorescence at the level of individual molecules, which displays intricate chemical transformations and, thus, provides deep and unique insights. Due to their high spatial and temporal resolutions, single‐molecule fluorescence microscopy and spectroscopy are also widely employed in various other fields, including biophysics,^[2]^ materials science^[3]^ and nanotechnology.^[4]^ Whereas at the very beginning of SMC, confocal microscopy was used to study the fate of individual molecules, imaging methods are now considered superior due to their ability to parallelize the observation of many molecules. Among these techniques, especially Total Internal Reflection Fluorescence (TIRF) microscopy is nowadays most commonly used, as it permits the analysis of processes at or near the surface of the sample, offering a high signal‐to‐background ratio. One convenient way to map for instance the heterogeneous turnover of special catalytically active sites on a surface employs the change of leuko‐dyes or dark molecules into fluorophores (fluorogenic reaction).[^5^, ^6^] Our approach, however, is based on the change of the electronic structure of a fluorophore,^[7]^ which, consequently, is called the participant approach.^[8]^ This approach demands tailored fluorophores which are mostly not commercially available and, consequently, is preceded by exhaustive syntheses.[^9^, ^10^] One exception is paradigmatic terrylene which promoted many scientific achievements in the early phase of single‐molecule fluorescence spectroscopy.[^11^, ^12^, ^13^, ^14^]

**Figure 1:**
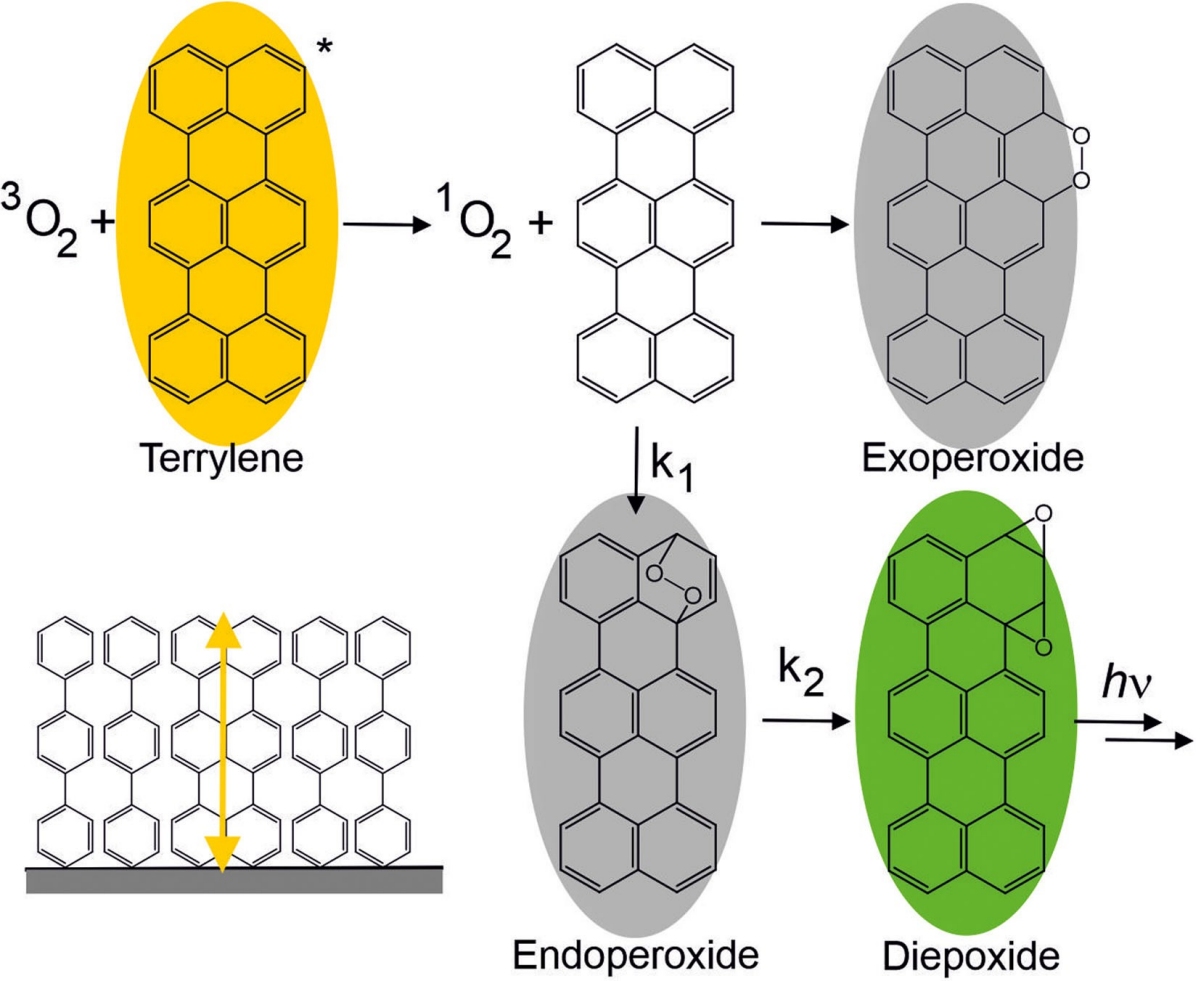
Suggested reaction mechanism between molecular oxygen in its triplet state and terrylene,^[15]^ yellow represents terrylene emission, green indicates the photoproduct luminescence, and grey denotes luminescence switch off. Bottom left: Scheme of embedded terrylene molecules in *p*‐terphenyl matrix. The yellow arrow indicates the orientation of the transition dipole moment perpendicular to the glass surface (grey).

Terrylene is even a well‐suited compound for single‐molecule fluorescence chemistry owing to its luminescent properties, photostability and its ability to be embedded in solid matrices.[^13^, ^15^] In a seminal work, Th. Basché and coworkers^[15]^ observed the conversion of terrylene into hypsochromically shifted photoproducts. The monitored reaction is, in a first step based on quantum chemical calculations, suggested to arise from triplet‐triplet energy transfer between the T_1_ state of terrylene and molecular oxygen forming singlet oxygen ^1^O_2_.^[15]^ Then, cycloaddition of ^1^O_2_ on the terrylene core results in the formation of exo‐ or endoperoxide products as putative main, dark products. Specifically, the endoperoxide can further decompose to generate a fluorescent diepoxide and other photoproducts with different luminescence spectral shifts relative to the terrylene spectrum. It should be mentioned that no analytical proof of the intermediate's chemical nature is available yet. Nevertheless, the proposed mechanism^[15]^ is illustrated in Figure 1.

The likelihood of producing a luminescent product, however, is low, and might be a sign of the preferred Diels‐Alder reactivity in the bay‐region of rylene dyes.^[16]^ Actually, 80 % of the molecules undergo an irreversible transition into a dark state, if bleaching takes place at all.^[15]^ Moreover, terrylene seems to be shielded from oxygen attack when deeply buried in the crystalline packing.

Whereas SMC experiments with terrylene are scarce, some studies with its derivative terrylene diimide (TDI) were conducted: reaction with externally produced ^1^O_2_ yielded blue‐shifted fluorescent products, similar to those products which were generated by the photooxidation in polymer films.[^17^, ^18^] These experiments were exhaustively modeled by quantum‐chemical computations which suggested even NIR‐luminescent photoproducts.^[19]^ As workhorse for our ongoing technical improvements in SMC, we reexplore the photoreaction mechanism of terrylene oxidation. In this study, we aim at providing rate constants and approximate quantum yields for the different processes along the reaction pathway from terrylene to its photoproducts which was cumbersome in the original experimental setting.^[15]^ Although the accepted reaction mechanism suggests an unimolecular, first‐order decomposition of the intermediate entailing a monoexponential distribution of intermediate survival time,^[20]^ we find an obvious deviation. A significant and unexpected effect of the excitation wavelength on the reaction mechanism points to an immediate passage after the first reaction step towards the final products.

## Results and Discussion

### Absorption and Luminescence Spectra

**Figure 2:**
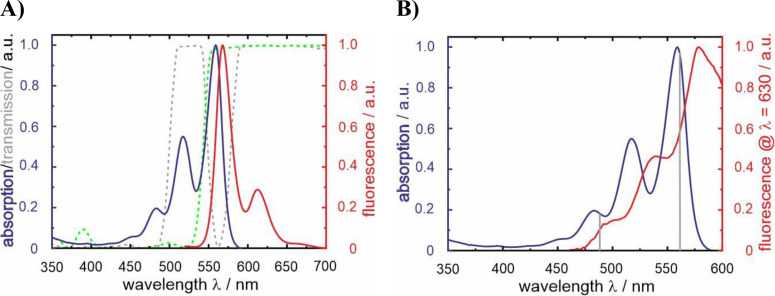
A) Normalized absorption and luminescence spectra of terrylene in toluene. The grey and the green spectra (dotted) represent the transmission spectra of the 488/561 nm dual‐edge beamsplitter and of the 543 nm dichroic mirror, respectively. B) Normalized excitation spectrum of terrylene in *p*‐terphenyl (red) in comparison to the normalized absorption spectrum of terrylene in toluene (blue). The grey lines in B indicate the excitation wavelengths used in the single‐molecule experiments.

The already established sample system of terrylene embedded in *p*‐terphenyl was investigated. In contrast to the original study,^[15]^ where terrylene was co‐sublimated with *p*‐terphenyl, we used spin‐coating for the sample preparation, following another preparation method,^[21]^ which gives smaller crystalline areas (see the experimental section for details). By this modification, oxygen access to terrylene is facilitated and, moreover, the orientation of the transition dipole moment, roughly perpendicular to the glass surface, allows for identifying the probe molecules in a proper environment. For characterization, the absorption and luminescence spectra of terrylene were measured in toluene (Figure 2A). The first electronic absorption band exhibits a vibronic fine structure with pronounced maxima located between λ_abs_=450 nm and 590 nm with a maximum at λ_abs_=559 nm. The vibrational spacing corresponds to a typical CC‐stretching mode of the carbon frame.^[22]^ The fluorescence spectrum is almost the mirror image of the first electronic absorption band with a Stokes shift of Δλ=9 nm as expected for a rather stiff molecular framework.

As scattering contributions from samples embedded in *p*‐terphenyl are not straightforward to correct for, only the excitation spectrum provides the absorption shift of 19 nm induced by the solid matrix (Figure 2B). Considering this bathochromic shift of the absorption band to a maximum at λ_exc_=578 nm and the fact that the fluorescence of terrylene in the *p*‐terphenyl matrix peaks also at λ_em_~578 nm[^23^, ^24^] indicate that the solid matrix imposes a significant rigidity onto the degrees of freedom of terrylene.

Due to the low solubility of terrylene, which makes it unsuitable for further spectroscopic characterization, we assume that its fluorescence quantum yield is comparable to that of 2,5,10,13‐tetra‐tert‐butylterrylene, which has been determined to be 0.7.^[25]^ A similar value has been determined at cryogenic temperatures.^[26]^

### Experimental Conditions and Observations

**Figure 3:**
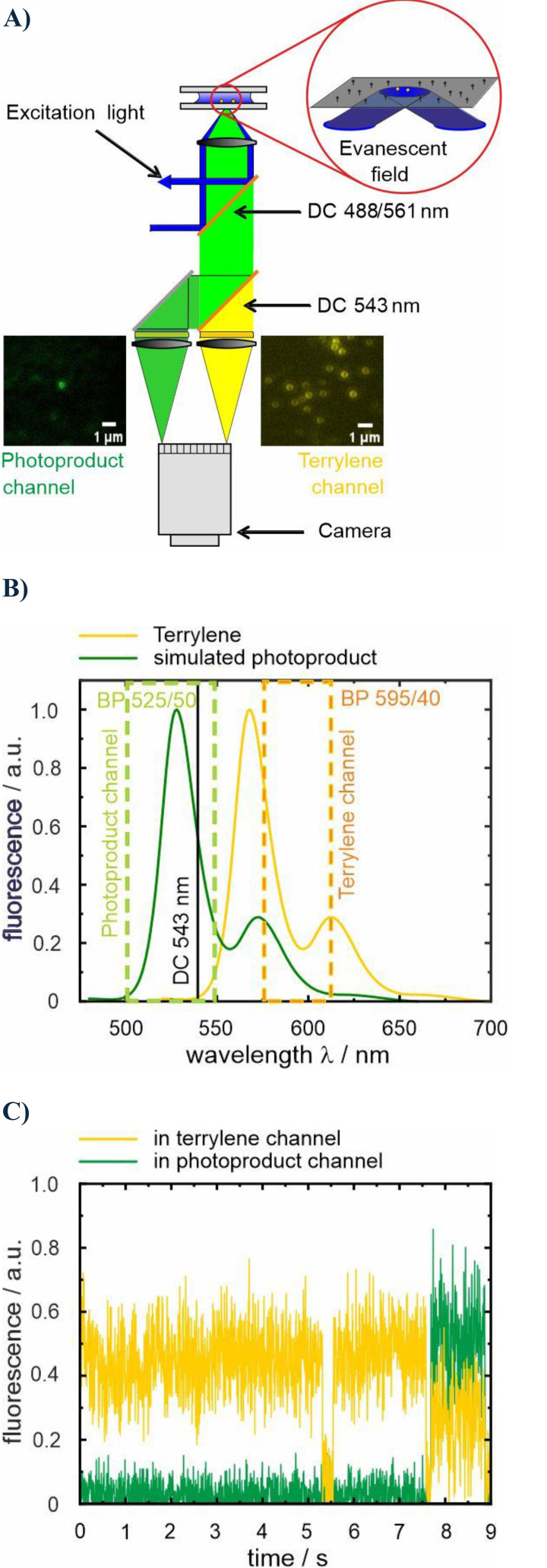
A) Scheme of TIRF setup. The brightest, unambiguously identified, reacting single molecule exhibits a count rate of ~73,000 detected photons s^−1^, resulting in a maximum overall detection efficiency of ~40 %. B) Range of wavelengths detected in the terrylene and photoproduct (based on a simulated spectrum, i. e., 40 nm blue shifted luminescence spectrum of terrylene resembling the most common product (total yield 7.6 %)^[15]^) channels. Product luminescence is partially detected in the terrylene channel. C) Luminescence time trace of a reacting terrylene molecule recorded with a time resolution of 5 ms.

The terrylene molecules were visualized using a home‐built objective‐type TIRF microscope, as depicted in the schematic in Figure 3A (see the experimental section for details). After excitation under total internal reflection conditions, the emission passes through a beam splitter (DC 543 nm, Figure 2A), enabling the visualization of terrylene and the photoproduct in two separate channels. The doughnut shape observed in the fluorescence of terrylene molecules (*cf*. Figure 3A) was attributed to the alignment of the terrylene emission dipole along the optical axis (z axis).^[27]^ In this configuration, the light distribution is rotationally symmetric with respect to the z axis, making it nearly independent of the azimuthal angle ϕ.^[28]^ Thus, only doughnut‐shaped fluorescent spots in the terrylene channel were analyzed as it verifies that single terrylene molecules in a crystalline environment were studied. To note, the emission spectrum of the main product is assumed to be blue‐shifted relative to that of terrylene.^[15]^ Therefore, a part of the product emission is detected in the terrylene channel (*cf*. Figure 3B), and the, presumably, much better excitability of the photoproduct at λ_exc_=488 nm (*cf*. Figure S1) leads to the observation, astonishing at first glance, that the photoproduct crosstalk in the terrylene channel can be much stronger than that of terrylene itself when only excited at λ_exc_=488 nm.

To elucidate the dependency of the terrylene photoreaction on the excitation wavelength, the system initially was excited at λ_exc_=488 nm with an estimated excitation intensity *I*_0_=3 kW/cm^2^ at the surface, thus, exciting both terrylene and the presumed main product.^[15]^ This is compared to the situation when terrylene is excited selectively at λ_exc_=561 nm (*I*_0_=0.6 kW/cm^2^), resulting in no luminescent spots in the green channel, as expected from the 40 nm blueshift of the photoproduct (*cf*. Figure S1). Therefore, the system was excited simultaneously at λ_exc_=561 nm and at λ_exc_=488 nm. For the latter, the excitation power was reduced by one order of magnitude compared to the first experimental setting, still enough to visualize the generation of photoproducts but, concomitantly, neglectable excitation of terrylene with vibrational excess energy. Due to the significant difference in terrylene's absorption coefficients at the excitation wavelengths (*cf*. Figure 2B), the excitation intensities were, thus, roughly adjusted to ensure a consistent excitation rate *k*_12_ for both parts of the study, according to the following equation:
(1)

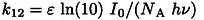




where ϵ is the absorption coefficient, N_A_ the Avogadro constant, h is the Planck constant, ν represents the light frequency and I_0_ is the intensity of the excitation light in units of kW/cm^2^. With ϵ=65,000 M^−1^ cm^−1^ at the excitation maximum,^[25]^ yielding ϵ=6580 M^−1^ cm^−1^ at λ_exc_=488 nm and ϵ=37570 M^−1^ cm^−1^ at λ_exc_=561 nm, respectively, the excitation rate is *k*_12_~1.8×10^5^ s^−1^ for the brightest molecules. This value represents an idealized upper limit, assuming perfectly parallel alignment between the fixed transition dipole moment of the molecules and the electric field of the excitation light, without accounting for different molecular orientations in the solid matrix.^[29]^

**Figure 4:**
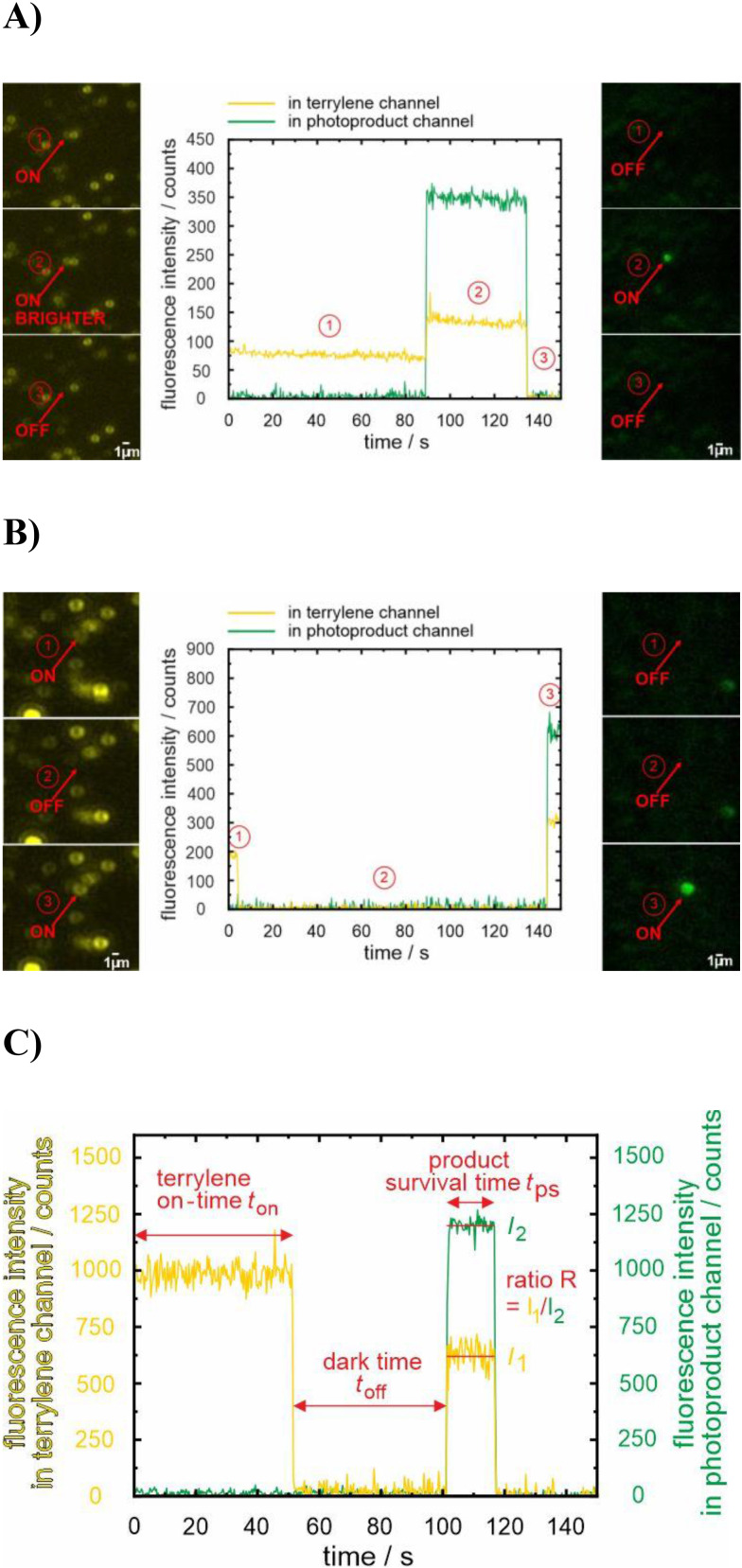
Single‐molecule events: A) without a dark time, B) with a dark time, observed through luminescence time traces and images recorded in the terrylene (yellow/orange) channel and the photoproduct channel (green) at λ_exc_=488 nm. C) Parameters extracted from the time trace of a reacting molecule: the terrylene on‐time *t*_on_, the reaction dark time *t*_off_, the product survival time *t*_ps_ and the ratio *R*.

Since the observation of the luminescent photoproduct is rare, approximately 70 movies of 150 s each at a time resolution of 0.3 s were recorded across 5 different samples to detect around 100 reacting molecules for each excitation condition. Please note that the excellent signal‐to‐noise and signal‐to‐background ratios made it possible to record reactions with a high time resolution down to 5 ms (see Figure 3C); however, the higher time resolution increases data storage requirements. Due to the limited computational capacity of our current setup, the higher time resolution entails recording smaller movie frame sizes with a considerably reduced number of reacting molecules and were therefore not used for the study here. Hence, each movie contains on average 400 terrylene molecules, though this number can vary depending on the specific spot, the sample, and the excitation wavelength. Despite the smaller dimensions of the prepared samples compared to the sublimated *p*‐terphenyl crystals of ref. [15], the majority of molecules seemed still to be protected by the crystalline packing from the reaction with oxygen. However, once a reaction is noticed, two distinct pathways could be observed (Figure 4), which are distinguished whether a ‘dark state’ is present or not (see the discussion below for consideration of t_off_=0 in exponential distributions).

### Analyses of Single‐molecule Data

Several parameters are extracted from the fluorescence time trace of each molecule for the two different experimental conditions (Figure 4C). The first parameter is the terrylene on‐time *t*_on_, which allows for confirming comparable excitation conditions for the two different wavelengths. The second and arguably the most important parameter is the dark time interval *t*_off_ between photobleaching of the terrylene molecule and the subsequent formation of the luminescent product. This period plays a pivotal role as it is intricately linked to the underlying photooxidation reaction mechanism.

**Figure 5:**
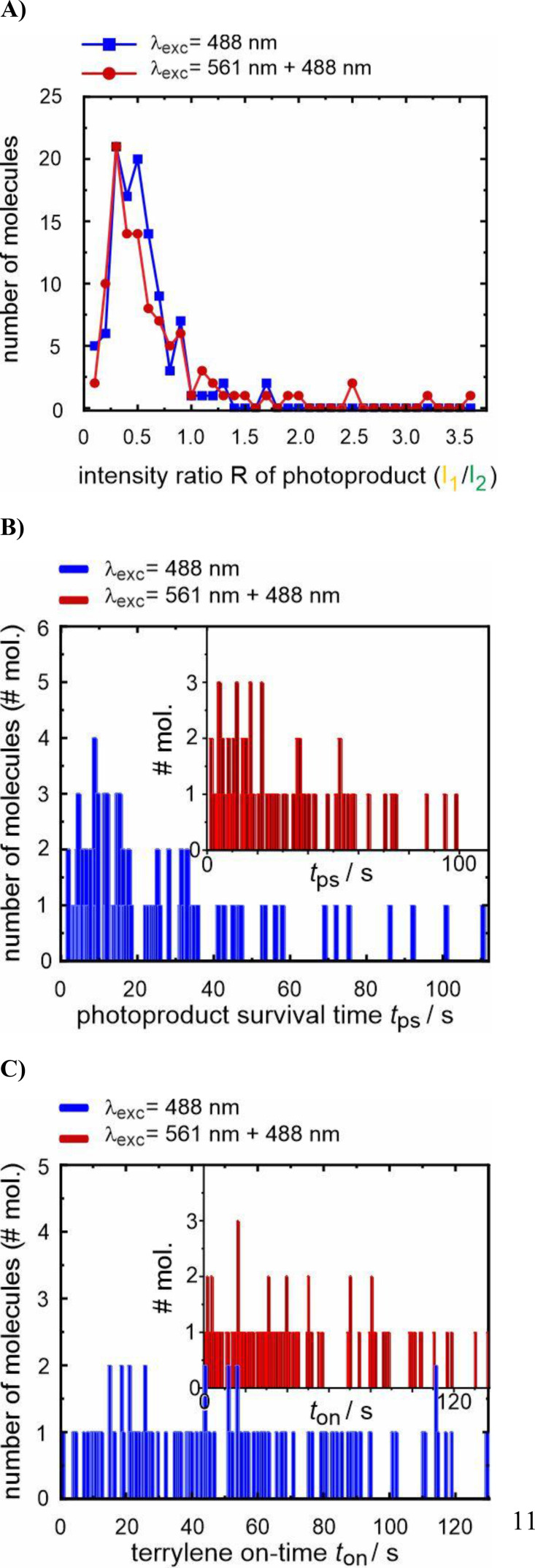
A) Distribution of ratios *R*, B) product survival time *t*_ps_, C) terrylene on‐time *t*_on_, with excitation at λ_exc_=488 nm (blue) and λ_exc_=561 nm and 488 nm simultaneously (red).

We start, however, our discussion with the analyses of the emission intensities and the survival time of the photoproduct, *t*_ps_, which both serve to superficially characterize the product variability. The ratio *R* of the photoproduct emission intensity detected in the orange channel, *I*_1_, to that detected in the green channel, *I*_2_, informs on potential differences in the chemical nature of the photoproducts. Comparing the distributions of the ratios for both excitation conditions (Figure 5A), both plots show a broad maximum close to *R*=0.3 which is interpreted as a similar product composition. This approach is rather crude, and the displayed distribution ratio might comprise different extents of hypsochromic shifts, as different photoproducts were detected in terrylene and TDI oxidation.[^15^, ^17^] Whereas a higher percentage of molecules with *R*~0.5–0.7 was found for λ_exc_=488 nm, a ratio>1 is more often met with λ_exc_=561 nm than with λ_exc_=488 nm, but still in the order of ~10 % and, presumably, of minor statistical significance. Whether this outcome indicates a preference for one photoproduct over the other will be investigated elsewhere using spectrally resolved data. It should also be emphasized that red‐shifted products are not detected in the current experimental setting.

The mean photoproduct survival time <*t*_ps_> (Figure 5B) for exciting at only λ_exc_=488 nm and λ_exc_=561 nm and 488 nm simultaneously yields values of (23.8±2.4) s and (28.1±2.7) s, respectively. For the latter excitation conditions, as only exciting at λ_exc_=488 nm allows for photoproduct excitation, the mean survival time <*t*_ps_> is expected to be considerably longer due to less photobleaching of the photoproduct, as the intensity for exciting at λ_exc_=488 nm is distinctly lower. Two biases with opposite impact on the determined photoproduct survival time *t*_ps_ should be mentioned: On the one hand, product molecules which did not switch off within the temporal observation window (see Figure 4B for an example) were excluded from this assessment, leading to an underestimation of <*t*_ps_>. This exclusion accounted for 14 % of the molecules excited at only λ_exc_=488 nm and 23 % of those excited at λ_exc_=561 nm and 488 nm simultaneously. On the other hand, we assume that the average lifetimes are overestimated, because shorter on‐times of the photoproduct *t*_ps_ may have been overlooked with a time resolution of 0.3 seconds. This could also explain why the exponential maximum in Figure 5B does not occur at or close to *t*_ps_=0. No correlation was found between the intensity ratio *R* of the photoproduct and its mean survival time <*t*_ps_> (*cf*. Figure S2). To summarize, the similarity of the distributions and the difference of the photoproduct survival times do not yet justify suggesting alternative outcomes of photoproduct distributions but deserve further investigations in the future.

Next, the terrylene mean on‐times <*t*_on_> (Figure 5C) are considered. As the reaction is presumed to be bimolecular, involving oxygen and terrylene, and the concentration of the latter being negligible compared to that of oxygen, the decay process is expected to show a pseudo‐first order exponential distribution when oxygen uniformly penetrates into *p*‐terphenyl. The terrylene mean survival times for the reacting molecules are <*t*_on_>=(51.7±3.2) s for exciting only at λ_exc_=488 nm and <*t*_on_>=(41.3±3.4) s for exciting at λ_exc_=561 nm and 488 nm simultaneously. The rather small deviation in <*t*_on_> suggests a uniform excitation rate *k*_12_ regardless of the considerable differences of the excitation conditions. Despite having chosen rather consistent excitation rates *k*_12_ at the main excitation wavelengths, the observed 20 % reduction in <*t*_on_> when exciting simultaneously at λ_exc_=561 nm and 488 nm is, at least partially, due to the additional excitation at λ_exc_=488 nm. Further, the better visibility of terrylene with λ_exc_=561 nm closer to the upper sample surface under TIRF conditions may also account for the survival time reduction. Nonetheless, the reaction quantum yield Φ_r_ may be calculated according to the following equation:
(2)

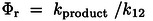




where *k*_product_ represents the rate constant for the formation of the photoproduct and *k*_12_ is the excitation rate as defined in Equation (1). The calculated quantum yields Φ_r_ are 1.07×10^−7^ for exciting only at λ_exc_=488 nm and 1.01×10^−7^ for exciting simultaneously at λ_exc_=561 nm and 488 nm. These values are on the same order of magnitude as determined for terrylene photobleaching in larger *p*‐terphenyl flakes.^[30]^ However, the vast majority of all reacting molecules is considerably dimmer than the brightest, reacting molecule which we used for estimating the detection efficiency (see caption Figure 3) by one order of magnitude (Figure S3). We attribute their weaker fluorescence intensity as a result of a larger distance from the glass/*p*‐terphenyl interface, reducing the excitation intensity of the evanescent field by a similar amount, and, therefore, a closer proximity to the upper crystal surface. If we take this experimental observation into account, a larger Φ_r_~10^−6^ is reasonable, closer to the value provided in ref. [23]. The unexpected finding of a similar Φ_r_ like in these publications may reflect that most terrylene molecules are still shielded from molecular oxygen despite the smaller dimensions of the prepared *p*‐terphenyl samples in this work. A closer inspection of the mechanism gives an explanation: as the concentration of terrylene in *p*‐terphenyl is so low, ^1^O_2_ sensitization can only occur with the individual terrylene molecule itself. For the spin‐allowed sensitization happening during the excited‐state lifetime of terrylene (τ_Fl_ ~4 ns, see below), O_2_ has to be very close or even in contact forming a complex with terrylene, whatever the diffusion coefficient of O_2_ in *p*‐terphenyl is. The intrinsic ISC rate constant, measured at cryogenic temperature, is extremely small^[31]^; at room temperature, however, the presence of molecular oxygen putatively enhances ISC.^[23]^ To note, almost every collision of ^3^O_2_ with an excited singlet‐state promotes ISC, however, the quantum yield for ^1^O_2_ formation is <1.^[32]^ This self‐sensitization mechanism is, therefore, in any case completely different to the reaction of TDI with ^1^O_2_, produced on a TiO_2_‐surface.^[18]^ The comparison of Φ_r_ with the quantum yield for intersystem crossing (ISC) Φ_isc_>10^−4^ (relying on the published lower value for the ISC rate constant *k*_isc_=2.7×10^4^ s^−1^ in *p*‐terphenyl^[23]^ and the fluorescence lifetimes τ_Fl_ in a polymer^[26]^) indicates that only a small fraction <1 % of ^1^O_2_ directly reacts with terrylene, whereas the vast majority may diffuse away without yielding photoproducts (for consideration of cycloreversion, see below).

**Figure 6:**
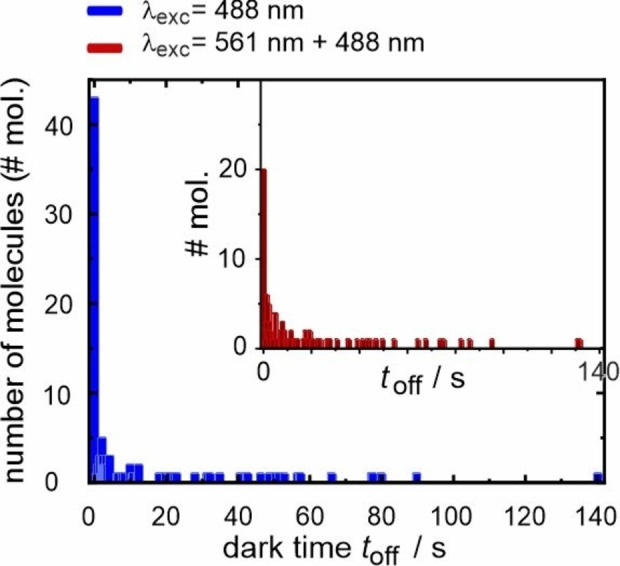
Statistics on dark times *t*_off_ with excitation of terrylene molecules at λ_exc_=488 nm (blue) or λ_exc_=561 nm and 488 nm simultaneously (red).

So far, the yet extracted data can be interpreted with a similar reaction mechanism for both excitation conditions. However, a disparity is observed in the population of molecules that transition directly to the photoproducts without a detectable dark time interval, *i. e*., *t*_1,off_=0 (Figure 6). Interestingly, 39 % of the molecules excited at λ_exc_=488 nm reacted without a dark time, which is twice as high as the 19 % observed for those excited simultaneously at λ_exc_=561 and 488 nm. Following a conventional kinetic approach for first‐order reactions, the decay is, at least, biexponential with lifetimes <*t*_1,off_> and <*t*_2,off_>, where the faster component is significantly shorter than the used time resolution of 0.3 s. Again, the time constant for each process (more concretely: for the slower process) is calculated as averaged dwell time.^[20]^

The mean values of the dark times for exciting at either λ_exc_=488 nm or at λ_exc_=561 nm and 488 nm simultaneously, excluding the molecules with *t*_1,off_=0, are comparable with values of <*t*_2,off_>=(23.2±3.4) s and (21.5±3.1) s, respectively. This quantity may characterize the stability of a relaxed endoperoxide as putative intermediate in the photooxidation reaction of terrylene. Taking the maximum constant for unimolecular reactions*, k*=10^13^ s^−1^, <*t*_2,off_> may be converted on the basis of a Boltzmann distribution to an activation energy *E*_A_ for the endoperoxide decomposition as high as 80 kJ mol^−1^ or 0.8 eV. This value is compared to quantum‐chemical data along the photooxidation reaction of the electron‐poor terrylene derivative TDI,^[19]^ where the decomposition of the intermediates towards the final products is activated by 80–100 kJ mol^−1^. From the reasonable agreement of *E*_A_, the reaction is interpreted to proceed via endoperoxide formation with the higher electron density in terrylene facilitating endoperoxide dissociation along the O−O bond with <*t*_2,off_>. Since cycloreversion as alternative pathway is a well‐known reaction mechanism for aromatic endoperoxides and a source of ^1^O_2_ generation,^[33]^ consideration of the high reversibility of the ^1^O_2_ attack is in line with the apparently low reaction yields mentioned above.

Considering also the appearance of a large proportion of trajectories with *t*_1,off_=0, this unambiguously shows the presence of an additional rapid process occurring beyond the used time‐resolution. This additional process may be attributed to an alternative reaction pathway such as ionization of terrylene and simultaneous formation of O_2_^−^, although at least for TDI oxidation no dependence on the excitation power was found,^[17]^ and excitation conditions in this work are considerably lower than those reported.^[15]^ Therefore, ultrafast transient absorption spectroscopy was used to shed light on the excited‐state dynamics.

### Transient Absorption Spectroscopy

**Figure 7:**
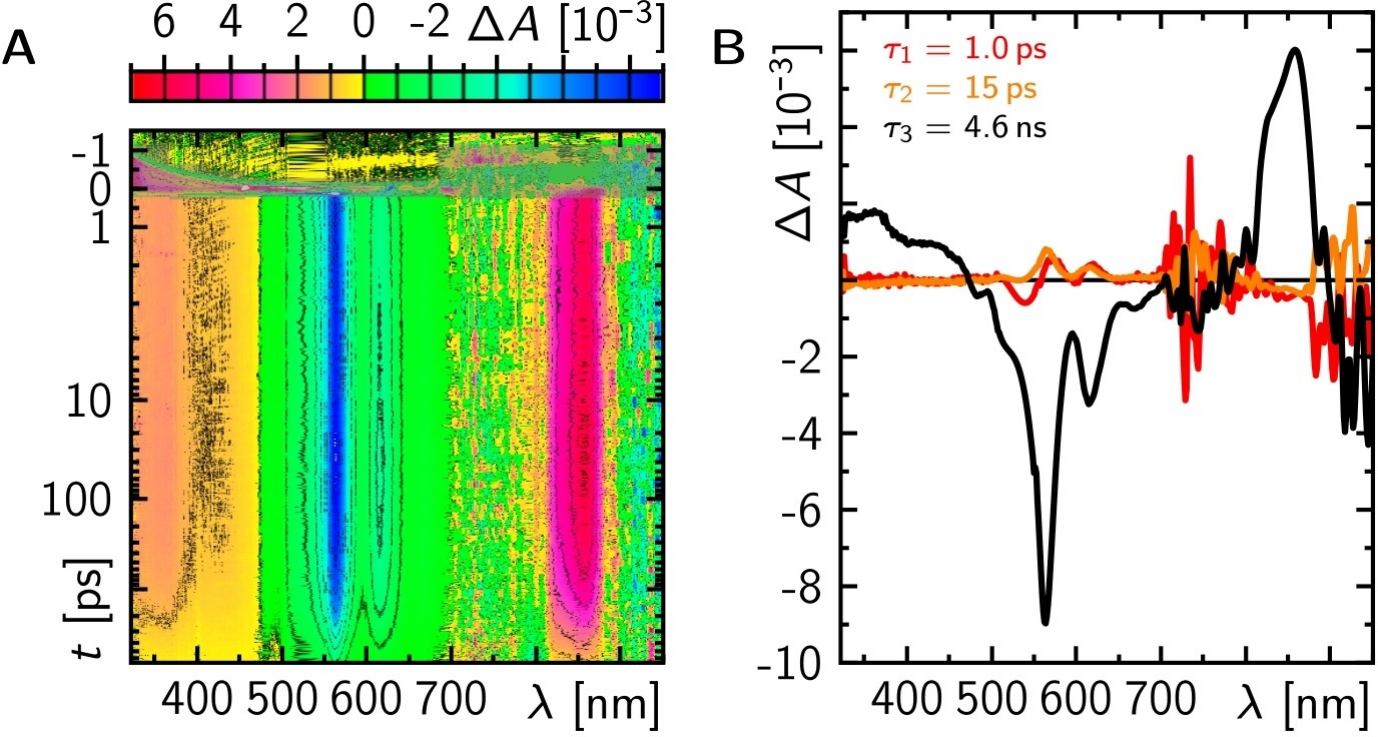
Transient absorption spectra of terrylene in toluene following a 100 fs excitation pulse at 530 nm (A). Grey areas around time zero indicate areas of the coherent artefacts that were patched during the global exponential fitting procedure as described in detail elsewhere.^[34]^ B) Decay associated difference spectra from three exponential global fit to the data in A.

The transient absorption of terrylene in liquid toluene at room temperature following ca. 100 fs excitation pulse at λ_exc_=530 nm reveals one rather narrow absorption band peaking at λ_probe_ ~850 nm and one rather broad absorption signal at λ_probe_ <490 nm intersected by the ground state bleach (GSB) and stimulated emission (SE) signals (Figure 7). The main positive spectral features correspond to the excited singlet state that decays accompanied by the simultaneous disappearing of the GSB and SE signals with a lifetime τ_FL_=4.6 ns (Figure 7)^[26]^ without the formation of detectable triplet absorption in line with Φ_ISC_ in the order of 10^−4^.

The main excited singlet decay is accompanied by intramolecular vibrational relaxation and redistribution (τ=1 ps) and solvent relaxation releasing excess heat to the surrounding (τ=15 ps)^[35]^ as evident in the slight modulations of the main spectral feature of the excited singlet state absorption without very dominant spectral features. The diffusional rotation time τ_rot_ of terrylene is 54.5 ps (Figure S4). Although the excited singlet state absorption feature at λ_probe_ <480 nm may be spectrally shifted in *p*‐terphenyl, its absorption surely does not exceed the cross section for GSB at λ_probe_=488 nm or 561 nm by orders of magnitude. As no strong excited singlet absorption at either excitation wavelength is observed, a re‐excitation of the excited singlet state to explain the fast component in the *t*_off_ distribution can be excluded. An alternative explanation, based on the available experimental findings, is required.

First of all, even a large fraction (~20 %) of terrylene molecules reacts without dark time when exciting at λ_exc_=561 nm close to the 0–0 transition at λ ~578 nm. Assuming identical reaction profiles of those reacting molecules with (<*t*_2,off_>) and without dark time (<*t*_1,off_>) by referring to the theoretical study on terrylenediimide TDI,^[19]^ and considering that any required energy in the reaction of terrylene with molecular oxygen to the diepoxide is only provided by light, then ^1^O_2_ formation (~1 eV) and passage of any overall activation energy (~1.5 eV; including the higher energy of the intermediate) exceeds already the provided excitation energy of 2.2 eV. Therefore, a common reaction path for both populations can be excluded.

To explain the fast process, the observed energy difference between λ_exc_=561 nm and 488 nm is taken into account, which amounts to another additional 0.3 eV or ~30 kJ mol^−1^. As self‐sensitization is the only way to initiate the photochemical reaction, we hypothesize that at least some terrylene/O_2_‐complexes adopt a favorable geometry for the reaction towards the intermediate and immediately react before any vibrational relaxation is completed. In fact, complexes of aromatics with oxygen are discussed for a long‐time.[^33^, ^36^] The excess energy from the excitation then is, partially, kept in this quasi‐unimolecular collision complex along its reaction path. A wavelength dependence of Φ_r_, implying higher vibrational excitation of the same electronic state, was observed also for other photochemical decompositions already long time ago,[^37^, ^38^, ^39^, ^40^] and shows that our explanation is not an unrealistic scenario for quasi‐unimolecular reactions.

However, there is one experimental finding which is puzzling: in none of the so far published studies on self‐sensitized single‐molecule photooxidation, <t_1,off_> =0 was observed. We trace this observation back to the weak statistics in previous studies. Altogether, the larger amount of molecules with <t_1,off_>=0 arises from a higher thermal contribution at λ_exc_=488 nm, allowing the molecules to overcome more easily any *E*_A_, necessary to generate the products via a different reaction mechanism directly from the excited singlet state and not via a relaxed, electronic ground state endoperoxide as intermediate. Whether another reaction mechanisms with an identical product distribution, e. g. [2+2] cycloadditions, play some role, cannot be decided on the basis of the current experiments.

## Conclusions

Studies on the reaction mechanism of terrylene and its derivatives are promising subjects for SMC as the substrate and, at least, some of its products are strongly fluorescing. In the present study, luminescence time traces of individual terrylene molecules in *p*‐terphenyl were exclusively analyzed showing self‐sensitized photooxidation to blue shifted photoproducts under light excitation at λ_exc_=488 nm and simultaneous excitation at λ_exc_=561 nm and 488 nm. On‐ and off‐times were analyzed on the basis of exponential distributions which appears justified due to the homogeneous environment. The reaction yield of Φ_r_>10^−7^ is independent of the excitation wavelength. The thermal stability of the putative intermediate endoperoxide is characterized by its survival time of <*t*_2,off_> ~20 s, which defines the activation energy *E*_A_ <80 kJ mol^−1^ for the decomposition towards the putative diepoxide as main photoproduct. External formation of ^1^O_2_ should lead to the same, relaxed intermediate,^[18]^ and should allow for verifying *E*_A_ in a study on the temperature dependence of the reaction as well as the yet unknown reaction barrier for the cycloreversion back to the starting material. Ongoing experiments will characterize the product range and are intended to investigate deviations among the different conditions. A higher time resolution and employing more advanced computational techniques including automized analyses will provide deeper insights, and low‐temperature emission spectroscopy may provide the vibrational signatures for an unambiguous product identification.

While the majority of reacting terrylene molecules undergo this widely accepted reaction mechanism, a minority may form photoproducts by bypassing the relaxed intermediate. It is tempting to speculate that this reaction follows a quasi‐unimolecular pathway within a pre‐assembled configuration with molecular oxygen starting from the excited singlet state of terrylene, with the stored excitation energy producing a hot intermediate, which then overcomes subsequent transition states with *E*_A_ ≪0.8 eV to the final photoproduct, a diepoxide. One requirement must be fulfilled for this mechanism to operate: the quasi‐unimolecular reaction itself must be faster than vibrational cooling. Indeed, apolar solvent relaxation of terrylene in toluene is relatively slow (τ=15 ps) and may be even slower in a crystalline environment. The hypothesis is supported by significant influence of the excitation wavelength on the reaction mechanism, as there is a substantial difference between the populations reacting without a dark time after excitation at λ_exc_=488 nm and at λ_exc_=561 nm and 488 nm simultaneously. A subsequent absorption of two photons could be excluded as the excited singlet state does not provide corresponding resonances for sequential two‐photon absorption. Whether excitation of higher vibrational quanta of a scaffold CC‐stretching mode in the Franck‐Condon region supports the reaction, remains rather speculative.

## Experimental Section

### Materials

Terrylene (99.5 % high purity, 10 mg) was purchased from Kentax, *p*‐terphenyl (≥99.5 % purity) and toluene (99.9 % purity) from Sigma‐Aldrich and used without further purification. The immersion oil (Immersol^TM^ 518 F) was bought from Zeiss.

### Sample Preparation

A 40 μL drop of a *p*‐terphenyl solution (2 mg/mL) in toluene containing a low concentration of terrylene (50 nM) was deposited on a glass coverslip (epredia, #1.5) using a home‐built spin‐coater. The coverslips themselves did not show any emission at the used excitation wavelength. This procedure provides thin films of terrylene embedded in the *p*‐terphenyl matrix with its transition dipole moment perpendicular to the glass surface. The molecular structure of *p*‐terphenyl is illustrated in **Figure** 1.

### TIRF Microscopy (see also Figure 3A)

The terrylene excitation was performed at λ_exc_=488 nm (at its maximum power of 38 mW) or simultaneously at λ_exc_=561 nm (7.6 mW) and 488 nm (3.85 mW) using a continuous‐wave (CW) laser (Toptica Photonics, iChrome FLE). After expansion of the diameter (GBE05‐A‐5X Achromatic Galilean Beam Expander, Thorlabs), the laser beams were focused into the back‐focal plane of the oil‐immersion objective (α‐Plan‐FLUAR, 100x, NA 1.45) on top of an inverted microscope (Axiovert 200, Zeiss), using an achromatic convex doublet lens (AC254‐400‐A‐ML, *f*=40 cm, Thorlabs). This lens allows to precisely control the spot at which the light hits the back aperture of the objective lense, and the beams, after being reflected by a dichroic mirror (dual‐edge beamsplitter 488/561 nm, AHF), hit the sample interface at an angle greater than the critical angle.^[41]^ This condition generates an evanescent field that penetrates the sample with an amplitude exponentially decreasing from the surface. The emitted light passes through the dichroic mirror before reaching another dichroic beamsplitter (DC 543, AHF) that splits the light into two beams; two bandpass filters (BP 595/40, AHF) and (BP 525/50, AHF) select the light of each beam forming two separate channels on the imaging detector, *i. e*., the terrylene channel and the photoproduct channel. Both channels were simultaneously visualized with the same low‐noise digital sCMOS camera (Orca fusion C14440, Hamamatsu). Movies featuring both channels were recorded, with a duration of 150 s each, at a rate of 3.34 frames per second and with a time resolution of 0.3 seconds. Data analysis and luminescence time traces were done using ImageJ software.

### Transient Absorption Spectroscopy

An in‐house build setup was used to cover the kinetics/dynamics on the sub‐ps to ns timescale as described previously.^[42]^ Here only the most relevant parameters are given: The terrylene sample dissolved in toluene (300 μL, OD≈0.05 over 1 mm at the excitation wavelength) located in a quartz cell (Starna) with 1 mm pathlength was excited at 530 nm into the S_1_←S_0_ electronic transition (pump pulse energy and temporal width of ca. 200 nJ and ca. 100 fs, respectively) and co‐linearly probed with a white‐light supercontinuum (WLSC, focussing a 800 nm pulse of ca. 1 mJ into a 5 mm thick CaF_2_ disc). The spot sizes for the pump and probe pulses at the sample position were adjusted to ca. 80 μm and ca. 30 μm, respectively. The sample was stirred by a magnetic stirrer bar rotating in the plane of the quartz cell driven by a rotating magnet to allow for sample exchange during the recordings. The time axis was chosen to be linear from −1 ps up to 4.0 ps in 20 fs steps and logarithmic afterwards, until the end of the delay stage of 6.5 ns. For a single scan 200 transient absorption spectra were recorded at each delay position. Each single spectrum was corrected for the dark current on the camera and for the fluctuations in the probe spectrum by referencing via a second camera prior to averaging. Averaging of 7 independent scans resulted in the final spectra. The polarization between pump and probe pulses was set to magic angle (54.73°) via a λ/2 plate in the pump beam path ensuring the recording of pure population dynamics of all excited states. The anisotropy of the transient absorption bands as for instance described in ref. [43] was determined by two subsequent measurements with setting the pump beam parallel (0°) and orthogonal (90°) to the probe beam, respectively. The averaged pre‐*t*_0_ laser scatter signal was subtracted from the data and the ca. 3 ps chirp of the WLSC is corrected for prior to data analysis using the coherent artefact as an indicator for time zero at each wavelength. Within the individual scans the data sets are identical. The integrity of the sample was checked by recording stationary absorption spectra before and after each measurement. No smoothing or filtering procedures were applied to the data.

## Conflict of Interests

The authors declare no conflict of interest.

1

## Supporting information

As a service to our authors and readers, this journal provides supporting information supplied by the authors. Such materials are peer reviewed and may be re‐organized for online delivery, but are not copy‐edited or typeset. Technical support issues arising from supporting information (other than missing files) should be addressed to the authors.

Supporting Information

Supporting Information

## Data Availability

The data that support the findings of this study are available in the supplementary material of this article.
