# Supplementary material for: Effect of Excitation Wavelength in Single‐Molecule Photochemistry of Terrylene
Source: Chemphyschem. 2025 Jan 26;26(7):e202400996. doi: 10.1002/cphc.202400996 (PMC11963977; doi:10.1002/cphc.202400996)
Supplement: Supplementary file 1 — Supporting Information [file CPHC-26-e202400996-s002.pdf]

ChemPhysChem

Supporting Information

Effect of Excitation Wavelength in Single-Molecule Photochemistry of Terrylene

Rana Mhanna, Julia Berger, Matthias Jourdain, Stephan Muth, Roger Jan Kutta, and Gregor Jung*

SUPPORTING INFORMATION

UNEXPECTED EFFECT OF EXCITATION WAVELENGTH IN SINGLE-MOLECULE PHOTOCHEMISTRY OF TERRYLENE

Rana Mhanna[1], Julia Berger[1], Matthias Jourdain[1], Stephan Muth[2], Roger Kutta[2], Gregor Jung*[1].

[1]*Department of Biophysical Chemistry, Saarland University, 66123 Saarbrücken, Germany.*
[2]*Institute of Physical and Theoretical Chemistry, University of Regensburg, 93053 Regensburg, Germany.*

Contents

Figure S1: Absorption spectrum of terrylene (orange) and simulated absorption spectrum of the photoproduct (green)…………………………………………………………………………………..2

Figure S2: Ratios as function of product photostability exciting at λ_{exc} = 488 nm (blue) and exciting at λ_{exc} = 561 nm and 488 nm simultaneously (red)…………………………………………………………………2

Figure S3: Terrylene on-times as function of molecules' brightness exciting at λ_{exc} = 488 nm (blue) and exciting at λ_{exc} = 561 nm and 488 nm simultaneously (red)…………………………………………………3

Figure S4 : Time-resolved anisotropy of the transient absorption following a 100 fs excitation pulse at 530 nm (**A**). **B**) Temporal slice along the data in **A**………..…………………………………………………….3

Visualization and time-resolved data of reacting molecules……………………………………………..4

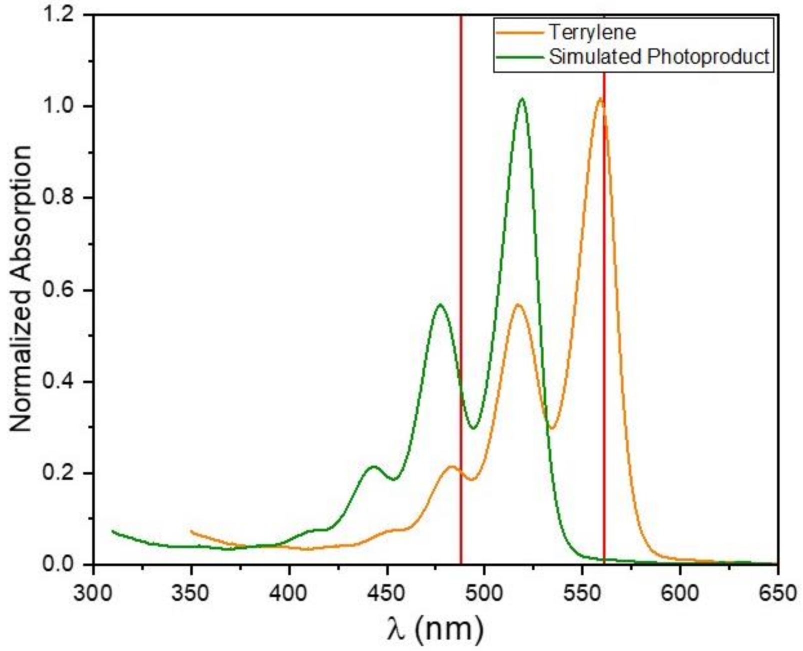

Figure S1: Absorption spectrum of terrylene (orange) and simulated absorption spectrum of the photoproduct[15] (green).

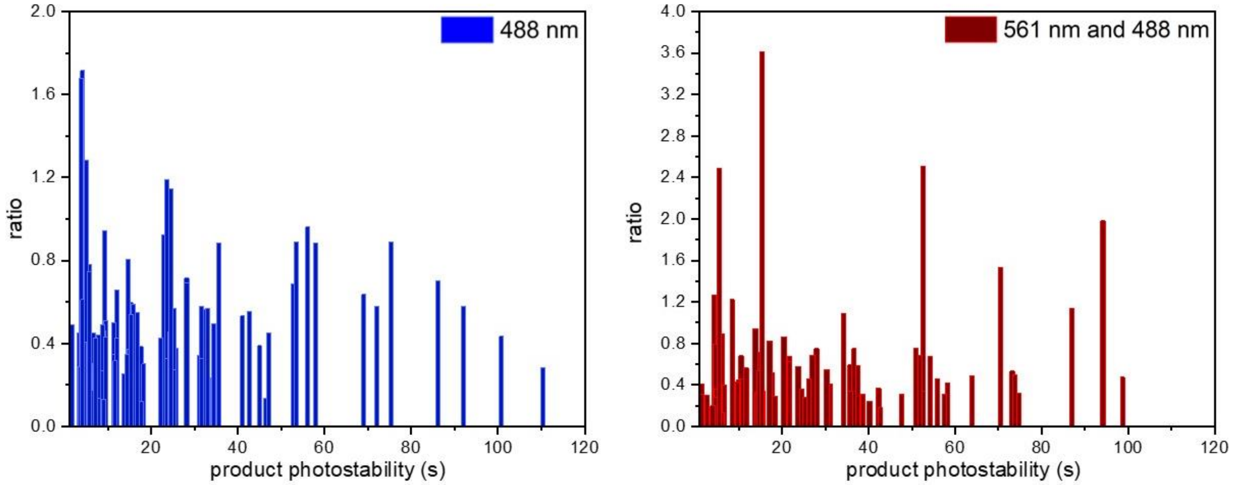

Figure S2: Ratios as function of product photostability exciting at λ_{exc} = 488 nm (blue) and exciting at λ_{exc} = 561 nm and 488 nm simultaneously (red).

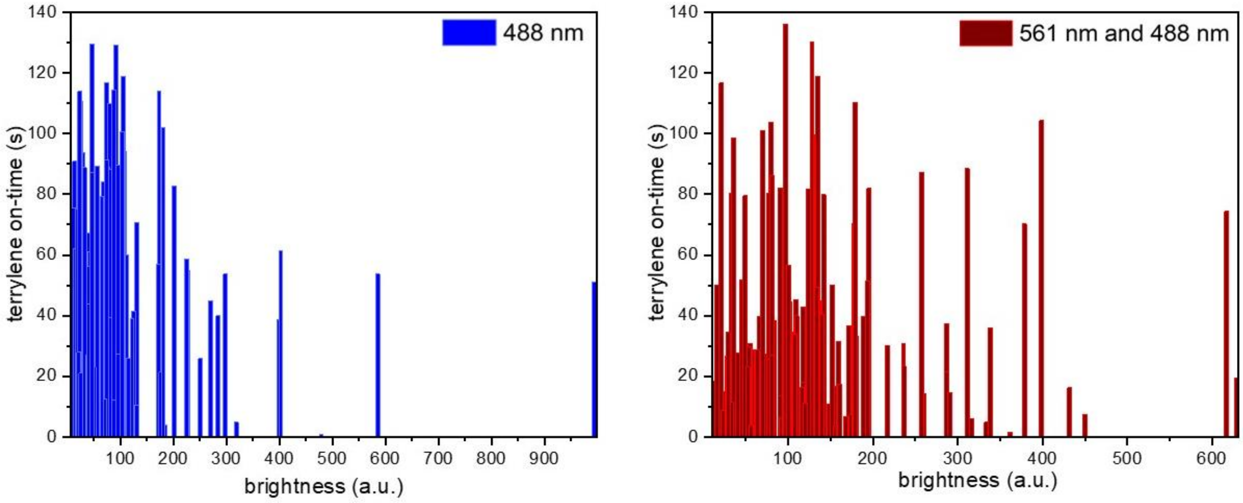

Figure S3: Terrylene on-times as function of molecules' brightness exciting at λ_{exc} = 488 nm (blue) and exciting at λ_{exc} = 561 nm and 488 nm simultaneously (red).

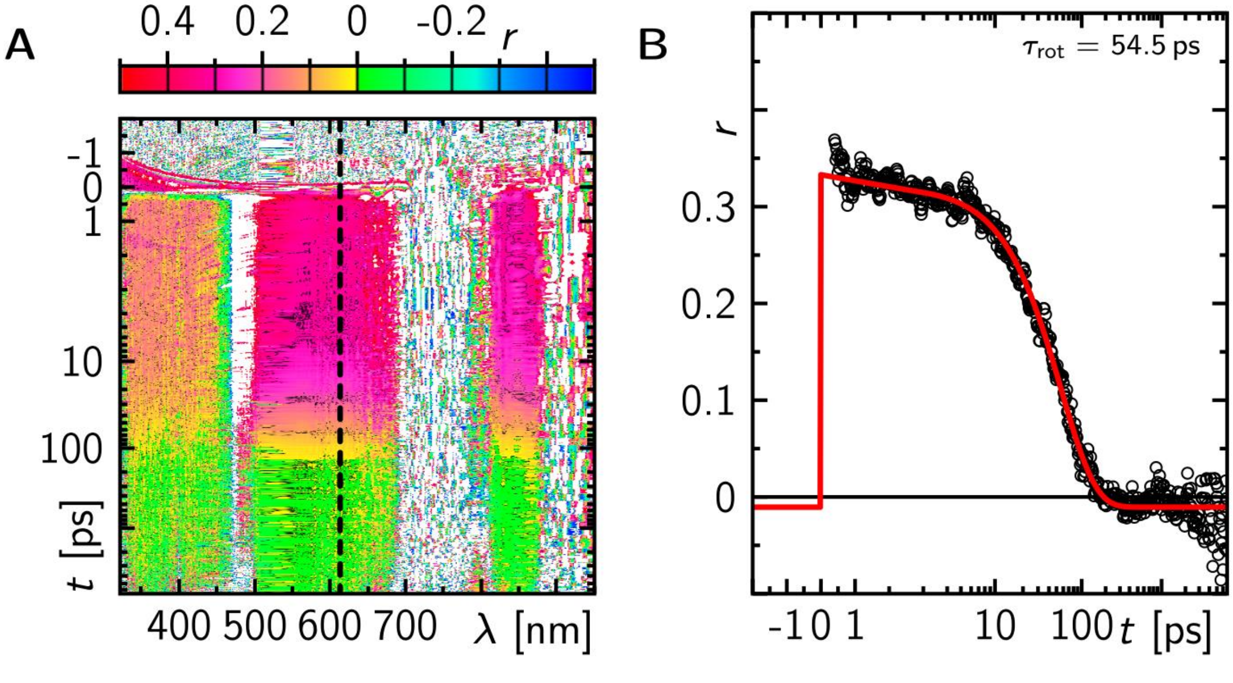

Figure S4: Time-resolved anisotropy of the transient absorption following a 100 fs excitation pulse at 530 nm (**A**). **B**) Temporal slice along the data in **A** as indicted by the dashed line including a mono-exponential fit to the data giving diffusional rotation time of 54.5 ps.

Visualization and time-resolved data of reacting molecules

1- Visualization of Reacting Molecules

A movie illustrating the two channels, terrylene channel (orange/yellow) and photoproduct channel (green), is provided. In this movie, three molecules that underwent reactions are highlighted by yellow/green arrows to demonstrate the processes visually. Molecules which are highlighted by a red arrow do not show the prototypical doughnut shape and were therefore excluded.

2- Time Trace Data

Time trace data of the reacting molecules are included for the two excitation conditions: λ_{exc} = 488 nm and λ_{exc} = 561 nm and 488 nm.

For each excitation condition, ~ 100 individual time traces are provided to ensure comprehensive representation of the dynamics observed.
